# Habitat selection and potential fitness consequences of two early‐successional species with differing life‐history strategies

**DOI:** 10.1002/ece3.5834

**Published:** 2019-11-19

**Authors:** Daniel Catlin, Daniel Gibson, Meryl J. Friedrich, Kelsi L. Hunt, Sarah M. Karpanty, James D. Fraser

**Affiliations:** ^1^ Department of Fish and Wildlife Conservation Virginia Tech Blacksburg Virginia

**Keywords:** demography, fitness, habitat selection, least tern, piping plover

## Abstract

Habitat selection and its relationship to fitness is a fundamental concept in ecology, but the mechanisms driving this connection are complex and difficult to detect. Despite the difficulties in understanding such intricate relationships, it is imperative that we study habitat selection and its relationship with fitness. We compared habitat selection of least terns (*Sternula antillarum*) and piping plovers (*Charadrius melodus*) on the Missouri River (2012–2014) to examine the consequences of those choices on nest and chick survival. We hypothesized that plovers and terns would select habitat that minimized the chance of flooding and predation of eggs, chicks, and adults, but that plovers would also select habitat that would provide foraging habitat for their chicks. We developed an integrated habitat selection model that assessed selection across multiple scales (sandbar and nest scales) and directly modeled the effect of selection on nest and chick survival. In general, the species selected habitat in keeping with our hypotheses, such that predation and flooding, in particular, may have been reduced. Sandbar selection had either a negative or no appreciable effect on nest survival for both species across years. Nest‐site selection in 2012 had a generally positive effect on nest survival and chick survival for both terns and plovers, and this trended toward a negative effect by 2014. This result suggested that early selection decisions appeared to be adaptive, but we speculate that relatively high site fidelity and habitat degradation led to reduced benefit over time. Our results highlight the complex nature of habitat selection and its relationship to fitness.

## Graphical abstract

While the connection between species and their habitats is broadly accepted, the connections between habitat and overall fitness often are less well understood. We compared habitat selection of piping plovers and least terns on the Missouri River (2012–2014) to examine the consequences of those choices on nest and chick survival. Our results suggest that early selection decisions were adaptive, but that relatively high site fidelity and habitat degradation led to reduced benefit over time.

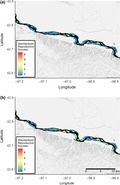

## 1 INTRODUCTION

Habitat loss is one of the most pervasive threats to biodiversity, affecting habitats and species around the globe (Hanski, 2011). Habitat selection and its relationship to underlying resources is a fundamental concept in ecology, conservation, and management (Boyce & McDonald, 1999). Though complex, the interplay between organisms and their habitat can teach us a great deal about selection and ecology (Southwood, 1977). While the connection between species and their habitats is broadly accepted (e.g., Elith & Leathwick, 2009), the effect of these connections on fitness often are less well understood. Anthropogenic changes in habitat, methodological issues, and complex ecological and evolutionary trade‐offs can obscure signals and result in mismatches between predicted fitness relationships and actual outcomes (Chalfoun & Schmidt, 2012). Continued comparative study, however, can aid in understanding the mechanisms behind these relationships.

Habitat selection is the product of a complex suite of selective pressures and behavioral choices, and perhaps nowhere is this complexity more evident than in the choice of a nest site for a bird. Adults choose a location that not only protects their reproductive investment, but often also their own safety. This choice can carry consequences for survival (Amat & Masero, 2004; Miller, Grand, Fondell, & Anthony, 2007), nest success (Murray & Best, 2014; Stokes & Boersma, 1998), and potentially fitness (Braden, McKernan, & Powell, 1997; Clark & Shutler, 1999; Martin, 1988; Orians & Wittenberger, 1991). Although nest‐site selection and its relationship with reproductive success is a widely researched topic (Jones, 2001), incongruences between selection and success are the norm and not the exception (Chalfoun & Schmidt, 2012). Despite this, knowledge of habitat selection can have important implications for guiding conservation and management, particularly for rare or sensitive species and in rapidly changing habitats.

Ground‐nesting birds and their nests are vulnerable to a variety of threats during the breeding season, and their choices reflect complex trade‐offs (Fraser & Catlin, 2019). Predation often is a primary cause of nest failure (Fletcher, Aebischer, Baines, Foster, & Hoodless, 2010; Smith, Gilchrist, & Smith, 2007), and the risk of predation may change with distance to predator habitat (Espie, Brigham, & James, 1996) and degree of concealment (Swaisgood et al., 2018), which may be evidenced in avoidance or preference for vegetative cover. For birds nesting near water, inundation also can cause nest failure (Espie et al., 1996), and nest‐site selection may reflect a balance between predator and flood avoidance (Greenberg et al., 2006; Storey, Montevecchi, Andrews, & Sims, 1988).

Individual nest success may not be the only selective pressure shaping selection. Thus, assuming random or maladaptive selection in the face of mismatched predictions may overlook other important fitness correlates (Chalfoun & Schmidt, 2012). For precocial birds whose chicks must feed themselves soon after hatch, proximity to foraging habitat can have a profound effect on habitat selection (Fraser & Catlin, 2019; Walker et al., 2019) and chick survival (Cohen, Houghton, & Fraser, 2009; Gibson, Blomberg, Atamian, & Sedinger, 2017; Loegering & Fraser, 1995), and these selective pressures must be weighed against other concerns (Chalfoun & Schmidt, 2012). Adult birds also must balance their own safety with that of their nests, and these trade‐offs may obscure the relationship between selection and fitness (Chalfoun & Schmidt, 2012; Gomez‐Serrano & Lopez‐Lopez, 2014; Guilherme, Burnside, Collar, & Dolman, 2018).

**Figure 1:**
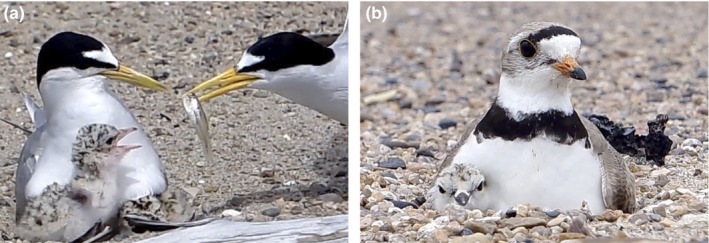
We studied habitat selection and its effects on nest and chick survival for (a) least terns (*Sternula antillarum*) and (b) piping plovers (*Charadrius melodus*) on the Missouri River (2012–2014)

Piping plovers (*Charadrius melodus*, “plovers”) and least terns (*Sternula antillarum*, “terns”; Figure 1) often nest together on Atlantic coast beaches (Burger, 1987) as well as on riverine sandbars in the Great Plains (Kruse, Higgins, & Lee, 2001). Both species generally select early‐successional, sparsely vegetated dry sand beaches to nest, where they dig small depressions in the sand to lay their eggs. While there have been numerous studies of piping plover nest‐site selection (e.g., Catlin, Fraser, Felio, & Cohen, 2011; Cohen et al., 2009; Espie et al., 1996; Gaines & Ryan, 1988), there have been relatively few for least terns (Kirsch, 1996; Kotliar & Burger, 1986; Sherfy, Stucker, & Buhl, 2012).

Despite the similarities in their ecology, there are key life‐history differences between the species. Plovers are territorial and their young are precocial, following their parents to wet sand feeding locations that the parents often defend from conspecifics (Catlin, Fraser, & Felio, 2015; Elliott‐Smith & Haig, 2004). These feeding territories have an important role in successfully raising a brood and how adults select nesting territories (Cohen et al., 2009; Loegering & Fraser, 1995; Walker et al., 2019). Terns, however, nest colonially, defend their colonies against predators, typically lay fewer eggs, and their young are semi‐altricial. Though the young are mobile soon after hatching, they rely on their parents to deliver them small fish until they are able to fish for themselves (Thompson et al., 1997). Terns also have a faster breeding cycle with a 21‐day nesting period and reaching flight at about 20 days, as opposed to plovers with a 34‐day nesting period and at least 25 days before flight (Catlin, Felio, & Fraser, 2013; Elliott‐Smith & Haig, 2004; Thompson et al., 1997). At least for plovers, the selection of a nest site can contribute to higher nest success in some cases (Espie et al., 1996; Prindiville‐Gaines & Ryan, 1988), but the relationship with chick survival is more uncertain (Cohen et al., 2009), and studies that directly relate habitat to fitness and studies that compare selection between the species are lacking. Given the similarities in gross habitat selection, one might assume that factors affect least terns similarly, but these critical tests are largely lacking, and differences between the way the two species select habitat and its effects on fitness could help our understanding of the benefits of habitat selection.

The objectives of this study were (a) to compare the second‐ and third‐order habitat selection of two, ground‐nesting species that use early‐successional habitats and (b) to develop a model that examines the consequences of those choices on nest and chick survival. To achieve this, we developed an integrated habitat selection model that assessed selection across multiple scales and directly modeled the effect of the strength of selection on nest and chick survival in plovers and terns. We examine how three potential hypotheses, avoiding predation, avoiding flooding, and optimizing foraging for adults and chicks, affect each species' choice of habitat and if it differs between them. To avoid predation of eggs, chicks, and themselves, we hypothesized that birds would avoid dense vegetation, perch trees, mainland sources of predators, and select habitat that maximized their crypsis (e.g., open, bare substrate; Fraser & Catlin, 2019). Flooding is a significant threat to seabirds and shorebirds (Espie et al., 1996; Sidle, Carlson, Kirsch, & Dinan, 1992), and thus, we hypothesized that birds would select habitat that minimized flooding (e.g., farther from the waterline, in dry rather than wet sand). The selection of habitat that fosters feeding of young is a key aspect to plover nest‐site selection (Walker et al., 2019), and thus, we hypothesized that plovers would select sandbars and nest sites that provided these opportunities (e.g., higher proportion of wet sand, nearer to wet sand). In particular, we were interested in how the differences in life‐history between plovers and terns would affect selection and how those differences in selection would affect reproductive success.

## 2 METHODS

### 2.1 Study area

**Figure 2:**
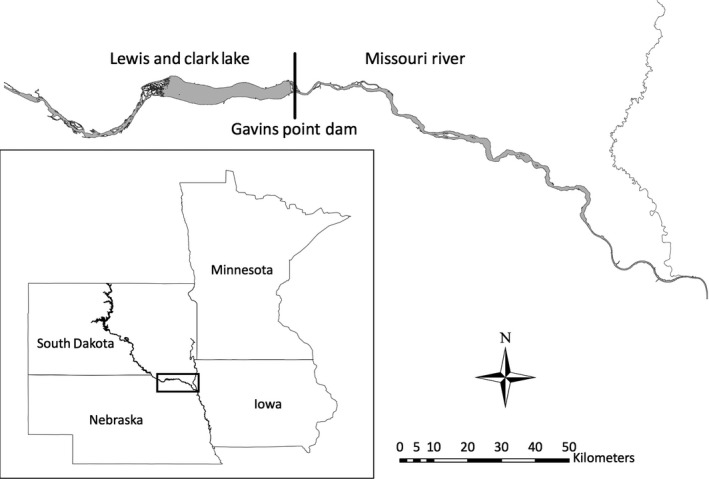
Map of the study area showing the location of the Gavins Point Reach between South Dakota and Nebraska, USA

The study took place in the Missouri National Recreational River on the Gavins Point Reach (GVP). GVP is a 95‐km stretch of river in South Dakota and Nebraska, USA, between the Gavins Point Dam (42°51′N, 97°29′W) and Ponca State Park (42°36′N, 96°42′W) in 2012–2014 (Figure 2). In 2011, historically high flows throughout the Missouri River system inundated much of the sandbar habitat on GVP (Catlin et al., 2015), with high water precluding nesting and territory establishment for both species on GVP. Following the flooding in 2011, however, there was a nearly 10‐fold increase in open and sparsely vegetated sand, and plovers and terns resumed breeding at these sites (Hunt, Fraser, Friedrich, Karpanty, & Catlin, 2018).

### 2.2 Field methods

We surveyed open and sparsely vegetated areas of emergent sandbars during the plover and tern breeding season (April–August) in search of nests. We located nests by grid‐searching potential nesting habitat, using spotting scopes to look for incubating birds, and recognizing behavioral cues of adult birds (e.g., territorial and distraction displays). Upon discovery, we logged each nest location with a handheld GPS unit (Trimble Geo XT, Trimble Navigation, Ltd.). Geographic coordinates had a horizontal accuracy of ±15 cm. We floated the eggs to estimate developmental stage (Westerskov, 1950) and calculate the expected hatch date. We attempted to check each nest every 2 days throughout the incubation period to determine nest fate, increasing our visit frequency within 3 days of the estimated hatch date when possible. If we observed ≥1 chicks or if ≥1 eggs disappeared within 2 days of the estimated hatch date without material evidence of failure (e.g., eggs washed out of nest bowl, predator tracks at nest, bloody eggshell), we considered a nest successful (Catlin et al., 2015; Hunt et al., 2018; Nefas, Hunt, Fraser, Karpanty, & Catlin, 2018). When no obvious signs of failure were present, but eggs disappeared >2 days before estimated hatch date, we considered the nest failed due to unknown cause. Common causes of plover and tern nest failure are depredation, flooding, weather‐related events such as heavy rain or hail, sandbar erosion, and abandonment. Potential nest predators in this region included raccoons (*Procyon lotor*), coyotes (*Canis latrans*), American crows (*Corvus brachyrhynchos*), and American mink (*Neovison vison*; Catlin et al., 2015; Catlin, Fraser, et al., 2011). Although the placement of wire predator exclosures (Melvin, Macivor, & Griffin, 1992) around plover nests is a common management practice to increase nest success (Johnson & Oring, 2002; Tan, Buchanan, Maguire, & Weston, 2015), none of the nests in this study were exclosed.

To assess chick survival, we banded all chicks with a unique combination of color bands (plovers) or a numbered, metal band (terns). We searched for these individuals approximately every 2 days until all birds would have been fledged or the end of the season. Plovers were resighted from a distance or recaptured (Hunt et al., 2018) to assess survival, but terns had to be physically recaptured to read their band. Common chick predators included the previously described suite of nest predators but also included predators such as great‐horned owls (*Bubo virginianus*; Catlin, Felio, & Fraser, 2011; Kruse et al., 2001). There had been some predator removal at these sites prior to the flooding in 2011 (Catlin, Felio, et al., 2011), but there was no predator control at these sites during the study.

### 2.3 Image collection and classification

Pan‐sharpened multispectral satellite imagery (5 m resolution) was collected each year (2012–2014) between April and October and classified using Definiens Developer Software. Each year, sandbars (defined as contiguous terrestrial habitat within the banks of the river) were digitized in the program eCognition, and the river was delineated from the floodplain (banks of the river channel), and habitats were classified by their vegetative cover (sparse <30%, low canopy >30%, and mid/high canopy >30%, presence of large perch trees for raptors and corvids), canopy height, and dryness of the substrate (L. Strong, U.S. Geological Survey, unpublished data).

### 2.4 Sandbar, nest, and random point attributes

We extracted habitat data related to each plover and tern sandbar (average value for the sandbar or minimum distance from any point on the sandbar, depending on the data type) and nest point from the classified land cover datasets. We assembled attributes that we predicted were related to predation (vegetation, isolation), flooding (wet sand, proximity to water), and foraging (proximity of wet sand and waterline; see Table 1 for all extracted values and specific hypothesized relationships with selection). The foraging hypothesis was specific to plovers, which have precocial young that need to feed themselves in situ soon after hatch. We did not have sufficient information on the quality of tern foraging habitats (open water) to perform similar analyses, but we did apply this hypothesis to tern sandbars and nest locations for comparison with the plover results. In each year, we sampled approximately five unused sandbars for each used sandbar and eight random nest locations for each used nest for use in our habitat selection models. We used ArcGIS 10.5 (ESRI) to generate random points and to derive habitat data from the classified imagery. There were no limitations placed on the selection of unused sandbars and random points other than that they were within the boundaries of the river and were not in open water. All habitat covariates were standardized prior to inclusion in the analyses.

**Table 1:** Variables used to estimate habitat selection at the sandbar and nest‐site scale for piping plovers and least terns, nesting on the Missouri River (2012–2014) and their relationship to predicted fitness correlates. In addition, we have summarized the mean and range for these variables across scales and species at used and unused sites. Mean ± 1 *SD*

Scale	Variable	Hypothesis	Tern^a^	Plover^b^	Unused^c^
Sandbar	Island versus pointbar	Predation (+)	67/84 ± 0.48	28/42 ± 0.40	351/588 ± 0.49
	Prop. wet sand	Flooding (−), foraging (+)	0.16 ± 0.15	0.15 ± 0.19	0.37 ± 0.43
	Prop. dry sand	Flooding (+), foraging (−)	0.60 ± 0.28	0.46 ± 0.25	0.10 ± 0.22
	Sandbar area (ha)	Predation (+), flooding (+)	34.3 ± 34.9	30.6 ± 29.7	23.0 ± 72.7
	Prop. low canopy cover	Predation (−)	0.04 ± 0.09	0.08 ± 0.11	0.05 ± 0.12
	Prop. moderate‐high canopy cover	Predation (−)^d^	0.04 ± 0.08	0.12 ± 0.17	0.21 ± 0.31
	Average river width (m)	Predation (+)	1,466 ± 355	1,399 ± 352	1,332 ± 393
	Distance to floodplain bank (m)	Predation (+)	430 ± 173	435 ± 183	297 ± 220
	Distance to cover (m)	Predation (+)	230 ± 139	129 ± 122	85 ± 99
	Distance to nearest tree (m)	Predation (+)	335 ± 173	233 ± 145	187 ± 178
Nest	River width (m)	Predation (+)	1,537 ± 335	1,467 ± 381	1,455 ± 374
	Distance to nearest cover (m)	Predation (+)	303 ± 127	214 ± 123	129 ± 137
	Distance to nearest tree (m)	Predation (+)	418 ± 189	315 ± 144	257 ± 211
	Distance to floodplain (m)	Predation (+)	468 ± 139	447 ± 168	399 ± 202
	Dry sand (Y/N)	Flooding (+)	411/424 ± 0.17	307/326 ± 0.23	3,632/5,457 ± 0.47
	Distance to wet sand (m)	Flooding (+), foraging (−)	144 ± 151	124 ± 123	225 ± 215
	Distance to waterline (m)	Flooding (+), foraging (−)	80 ± 56	84 ± 56	113 ± 143

a Sandbars that were used by both terns and plovers (*n* = 84) and tern nesting sites (*n* = 424). At the sandbar scale, there were few sandbars (*n* = 6) with only tern nests, so we lumped them with sandbars that had both tern and plover nesting for analysis.

b Sandbars that only were used by plovers (*n* = 42) and plover nesting sites (*n* = 326).

c Sandbars that were used by neither terns nor plovers (*n* = 588) and randomly selected, unused nest sites (*n* = 5,457).

d Each factor was associated with three broad categories, habitat selection that reduces (a) predation and (b) flooding, (c) or provides access to foraging (plovers only, but terns were analyzed for comparison). For each, “+” indicates a hypothesis that the factor is positively correlated with selection, and “−” indicates negative correlation.

### 2.5 Model

We developed an integrated habitat selection model that tied second‐ (sandbar) and third‐order (nest‐site) selection and their predicted values to a model of nest success and a model of chick survival. For sandbar (*i*) selection, we modeled use as a multinomial, where the outcomes (*j*) were: (a) only used by plovers, (b) used by both plovers and terns, and (c) unused. Very few sandbars were only used by terns (*n* = 6) so we lumped these into the second category for analysis. Categories 1 and 2 contributed to selection for plovers, while only category 2 contributed to selection for terns. Thus, all sandbars chosen by plovers (regardless of tern behavior) were used to estimate plover sandbar selection. Likewise, only sandbars that terns selected (regardless of plover behavior) were used to estimate tern selection. For ease of description, we will refer to tern sandbars (i.e., used by both terns and plovers, but estimates for tern site selection only) and plover sandbars (only used by plovers).

$${\text{use}}_{i}∼\text{catergorical}\left(p_{i,j}\right)$$

$$\text{sandbar}\;{\text{use}}_{i,1}=p_{i,2}$$

$$\text{sandbar}\;{\text{use}}_{i,2}=1-p_{i,3}$$

We used our data to estimate year (y) and habitat‐specific probabilities of sandbar occupancy. Each covariate coefficient (*β_j_*_,_ *_x_*) was assigned a diffuse normal distribution, centered at 0, and all covariates appeared in a single, global model (see Appendix S1 for code).

$$\text{sandbar}\;{\text{selection}}_{i}=\alpha_{j,y}+\sum_{x=1}^{n}\beta_{j,x}\times {\text{covariate}}_{j,x}$$

$$\text{logit}\left(p_{i,j}\right)=\text{sandbar}\;{\text{selection}}_{i}$$

$$\alpha_{j,y}∼\text{logistic}\left(0,\;1\right)$$

$$\beta_{j,x}∼\text{normal}\left(0,\;1,000\right)$$

Then for each random and nest location (*k*), we modeled the probability that a specific nesting location was used as the joint probability that the location was a suitable nest site (*q_k_*) and that the probability that the sandbar was occupied by the species (*m*).

$$\text{nest}\;{\text{use}}_{k}∼\text{Bernoulli}\left(q_{k}\times \text{sandbar}\;{\text{use}}_{i,m}\right)$$

As with the sandbar selection model, we used normal distributions for priors on the habitat coefficients (*γ_x_*_,_ *_m_*) to identify important parameters.

$$\text{nest}\;{\text{selection}}_{k}=\sum_{x=1}^{n}\gamma_{x,m}\times {\text{covariate}}_{x,m}$$

$$\text{logit}\left(q_{k}\right)=\text{nest}\;{\text{selection}}_{k}$$

$$\gamma_{x,j}∼\text{normal}\left(0,\;1,000\right)$$

To estimate the effect of yearly sandbar and nest‐site selection on daily nest survival and thus nest success, we used a year and species‐specific logistic exposure model (Catlin et al., 2015; Hunt et al., 2018; Rotella, Taper, & Hansen, 2000; Shaffer, 2004). We chose to use the combined selection coefficients from the second‐ and third‐order selection models rather than the specific habitat variables because such relationships often are complex, and correlation and misspecification can contribute to failures to detect associations between selection and success (Chalfoun & Schmidt, 2012). We used a standard, diffuse normal prior for the effects of sandbar and nest‐site selection.

$$\text{daily}\;\text{nest}\;{\text{survival}}_{i,m,k,y}∼\text{Bernoulli}\left(s.{\text{nest}}_{i,m,k,y}\right)$$

$$\text{logit}\left(s.{\text{nest}}_{i,m,k,y}\right)=\delta_{0_{m,y}}+\delta_{1}\times \text{sandbar}\;{\text{selection}}_{i}+\delta_{2}\times \text{nest}\;{\text{selection}}_{k}$$

$$\delta_{0_{m,y}}∼\text{logistic}\left(0,\;1\right)$$

$$\delta_{1:2}∼\text{normal}\left(0,\;1,000\right)$$

For chick survival, we used a state‐space Cormack–Jolly–Seber model (Kéry & Schaub, 2012) to estimate daily age‐specific chick survival, whose format was essentially the same as for nest success except that we modeled the effect of age (*a*, days since hatch):

$$\text{logit}\left(s.{\text{chick}}_{i,m,k,y,a}\right)=\varepsilon_{0_{m,y}}+\varepsilon_{1_{m}}\times \left(a-1\right)+\varepsilon_{2}\times \text{sandbar}\;{\text{selection}}_{i}+\varepsilon_{3}\times \text{nest}\;{\text{selection}}_{k}$$

$$\varepsilon_{0_{m,y}}∼\text{logistic}\left(0,\;1\right)$$

$$\varepsilon_{1:3}∼\text{normal}\left(0,\;1,000\right)$$

$$\text{logit}\left({\text{resight}}_{i,m,a}\right)=\tau_{0:m}+\tau_{1:m}\times \left(a-1\right)$$

$$\tau ∼\text{normal}\left(0,\;1,000\right)$$

Because of sparse recapture data for terns, we set the age‐specific parameters to 0 for that species (i.e., survival and resight did not depend on age for terns). For full details of model specification, see Appendix S1. In addition to estimating nest and chick survival, we derived an overall measure of predicted reproductive success as the product of predicted occupancy (sandbar and nest‐site selection), nest success, and chick survival, which was standardized for comparison and can be projected over the habitat surface.

### 2.6 Model specification

We specified models within R (R Core Team, 2012) using the package “jagsUI” to call JAGS (Plummer, 2003) and export model results back to R. After assessing the performance of a series of exploratory model runs, we ran five chains of 201,000 with an adaptive phase of 1,000 runs and a burn‐in period of 1,000 iterations, thinning by 10 for 100,000 samples from the posterior distribution. We determined parameter convergence using the Brooks–Gelman–Rubin criterion ($\hat{R}$) (Brooks & Gelman, 1998) and by examining posterior plots, and we considered the model converged if it had an $\hat{R}$ < 1.1 at each parameter node across the entire nested model. We used effect size, the standard deviation of the posterior, and the proportion of the posterior on one side of 0 (“*f*”) to assess each parameter.

## 3 RESULTS

We collected information on habitat and bird use at 714 sandbar‐year combinations. Of those, 84 were used by both plovers and terns, 42 by plovers only, and 588 were unused. We monitored 424 tern nests and 326 plover nests, and we collected information on 5,457 randomly generated, unused locations. Apparent nest success was high, we only recorded 64 nest failures for terns (15.7%) and 67 nest failures for plovers (20.8%). In addition to monitoring nests, we captured and uniquely marked 537 tern chicks and 723 piping plover chicks across these sandbars.

### 3.1 Sandbar selection

**Figure 3:**
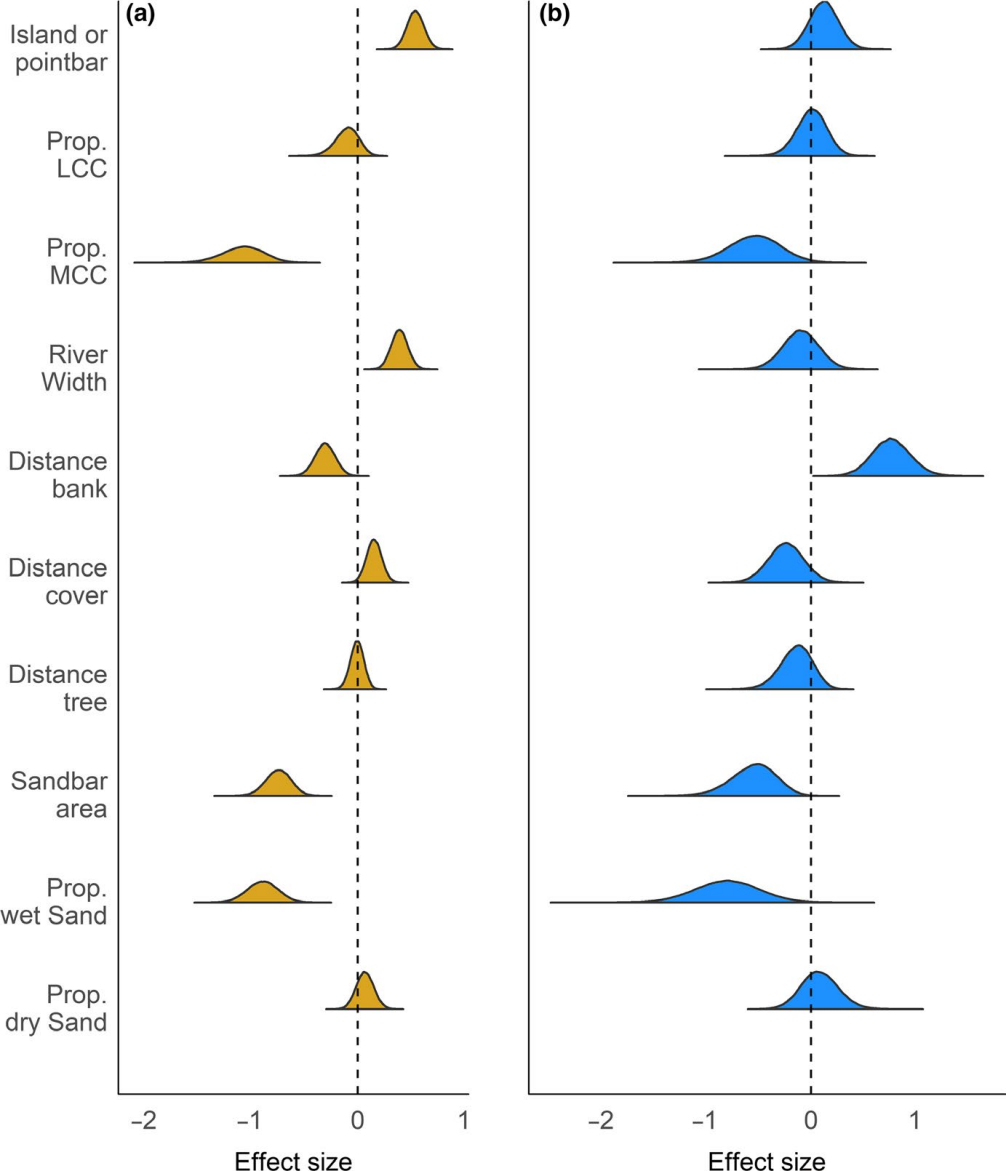
Sandbar scale selection coefficients for sandbars occupied by (a) least terns (*Sternula antillarum,* goldenrod) and piping plovers (*Charadrius melodus*) and (b) only piping plovers (blue) on the Missouri River. The estimates were derived from a multinomial regression, comparing these categories to unused sandbars. Negative and positive values indicate selection against or for, respectively, a factor relative to unoccupied sandbars. The dashed line indicates the origin, or no effect

Sandbars varied in their characteristics (Table 1). Our modeling indicated that plover and tern sandbars shared similar habitat characteristics (Figure 3), and their selection agreed with our hypotheses in general, particularly regarding predator and flooding avoidance (Table 2). For predation, both plover and tern sandbars tended to be islands rather than connected sandbars and they had a lower proportion of moderate canopy cover (Figure 3, Table 2). Tern sandbars also were in wider sections of the river, had a lower proportion of low canopy cover, and were farther from the nearest cover than unselected sandbars, though they also tended to be closer to the bank than unselected bars, the opposite of plover sandbars (Figure 3, Table 2). Plover sandbars tended to be farther from the nearest trees than unused bars, but tern sandbars were not different (Figure 3, Table 2). Both types of occupied sandbars tended to be smaller than unused bars, contrary to our hypothesis for both predator and flood avoidance. Both tern and plover sandbars, however, were composed of less wet sand and tended to have more dry sand than unused bars, in support of our flooding hypothesis, but not in line with our plover foraging hypothesis (Figure 3, Table 2).

**Table 2:** Parameter estimates from a Bayesian multi‐step regression analysis of habitat selection at the sandbar and nest‐site scale for piping plovers and least terns, nesting on the Missouri River (2012–2014) and their relationship to predicted fitness correlates

Scale	Variable	Hypothesis	Tern^a^	Plover^b^
Sandbar	Island versus pointbar	Predation (+)	0.54 ± 0.08 (1.00)	0.12 ± 0.14 (0.82)
	Prop. low canopy cover	Predation (−)	−0.10 ± 0.11 (0.83)	0.01 ± 0.14 (0.53)
	Prop. moderate‐high canopy cover	Predation (−)^c^	−1.08 ± 0.20 (1.00)	−0.54 ± 0.25 (0.99)
	River width (m)	Predation (+)	0.39 ± 0.08 (1.00)	−0.10 ± 0.17 (0.71)
	Distance to floodplain bank (m)	Predation (+)	−0.31 ± 0.10 (1.00)	0.77 ± 0.18 (1.00)
	Distance to cover (m)	Predation (+)	0.15 ± 0.07 (0.98)	−0.24 ± 0.17 (0.92)
	Distance to nearest tree (m)	Predation (+)	−0.01 ± 0.06 (0.54)	−0.14 ± 0.15 (0.82)
	Sandbar area (ha)	Predation (+), flooding (+)	−0.75 ± 0.12 (1.00)	−0.55 ± 0.21 (1.00)
	Prop. wet sand	Flooding (−), foraging (+)	−0.90 ± 0.15 (1.00)	−0.81 ± 0.31 (1.00)
	Prop. dry sand	Flooding (+), foraging (−)	0.07 ± 0.08 (0.79)	0.09 ± 0.18 (0.68)
Nest	River width (m)	Predation (+)	0.04 ± 0.42 (0.54)	−0.33 ± 0.20 (0.96)
	Distance to nearest cover (m)	Predation (+)	1.58 ± 0.96 (0.97)	0.24 ± 0.43 (0.68)
	Distance to nearest tree (m)	Predation (+)	5.04 ± 1.04 (1.00)	1.53 ± 0.35 (1.00)
	Distance to floodplain (m)	Predation (+)	−1.27 ± 0.67 (0.97)	−0.09 ± 0.24 (0.66)
	Dry sand (Y/N)	Flooding (+)	1.94 ± 0.56 (1.00)	0.54 ± 0.19 (1.00)
	Distance to wet sand (m)	Flooding (+), foraging (−)	−3.25 ± 0.72 (1.00)	−1.81 ± 0.31 (1.00)
	Distance to waterline (m)	Flooding (+), foraging (−)	2.11 ± 0.72 (1.00)	1.11 ± 0.36 (1.00)

Note

All variables were standardized prior to analysis so that effect sizes could be compared across estimates. For each estimate, we provide mean ± 1 *SD* from the posterior as well as the *f* value, or proportion of the posterior on one side of 0 in parentheses.

a Sandbars that were used by both terns and plovers (*n* = 84) and tern nesting sites (*n* = 424). At the sandbar scale, there were few sandbars (*n* = 6) with only tern nests, so we lumped them with sandbars that had both tern and plover nesting for analysis.

b Sandbars that only were used by plovers (*n* = 42) and plover nesting sites (*n* = 326).

c Each factor was associated with three broad categories, habitat selection that reduces (a) predation and (b) flooding, (c) or provides access to foraging (plovers only, but terns were analyzed for comparison). For each, “+” indicates a hypothesis that the factor is positively correlated with selection, and “−” indicates negative correlation.

### 3.2 Nest‐site selection

**Figure 4:**
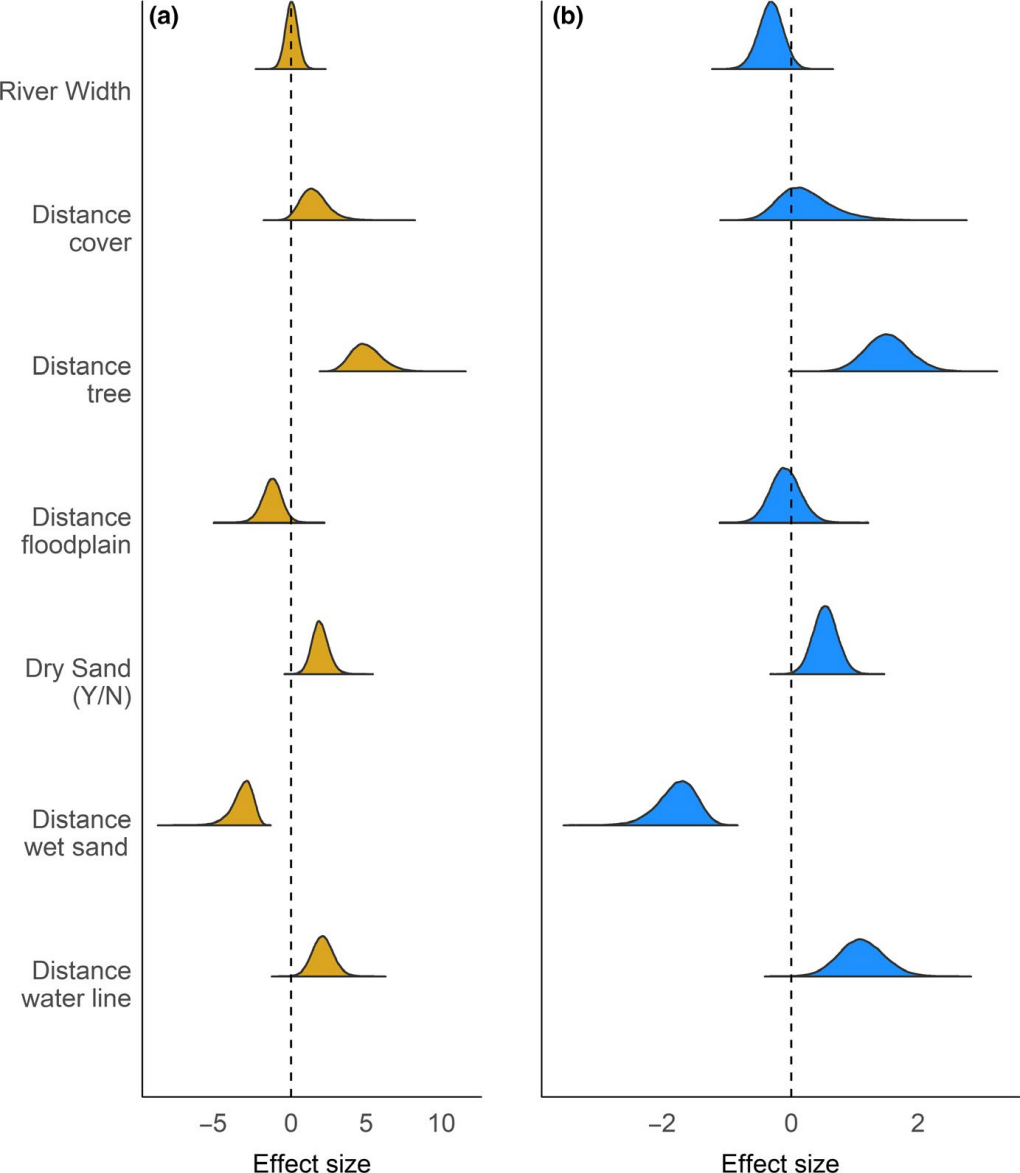
Nest‐site selection coefficients for nest sites occupied by (a) least terns (*Sternula antillarum*, goldenrod) and (b) piping plovers (*Charadrius melodus*, blue) on the Missouri River. The estimates were derived from a species‐specific logistic regression, comparing to unused sites. Negative and positive values indicate selection against or for, respectively, a factor relative to unoccupied sites. The dashed line indicates the origin, or no effect

Plovers and terns also used similar habitat characteristics when selecting their nest sites (Table 1). In general, plovers and terns selected habitat in keeping with our hypotheses relative to predator and flooding avoidance and that could optimize foraging, relative to unused sites (Figure 4, Table 2). For the predation hypothesis, nests of both species were farther from trees and terns were farther from cover than randomly selected sites, but they also tended to be closer to the flood plain than the random locations (Figure 4, Table 2). In terms of flooding, both species were more likely to nest in dry sand and farther from the waterline than random locations, but both species also were closer to wet sand than would be expected randomly, which agrees with the foraging hypothesis for plovers (Figure 4, Table 2). Overall, the strength of effect (magnitude of effect size) was greater for terns than for plovers (Figure 4, Table 2).

### 3.3 Effect of habitat selection on nest success and chick survival

**Figure 5:**
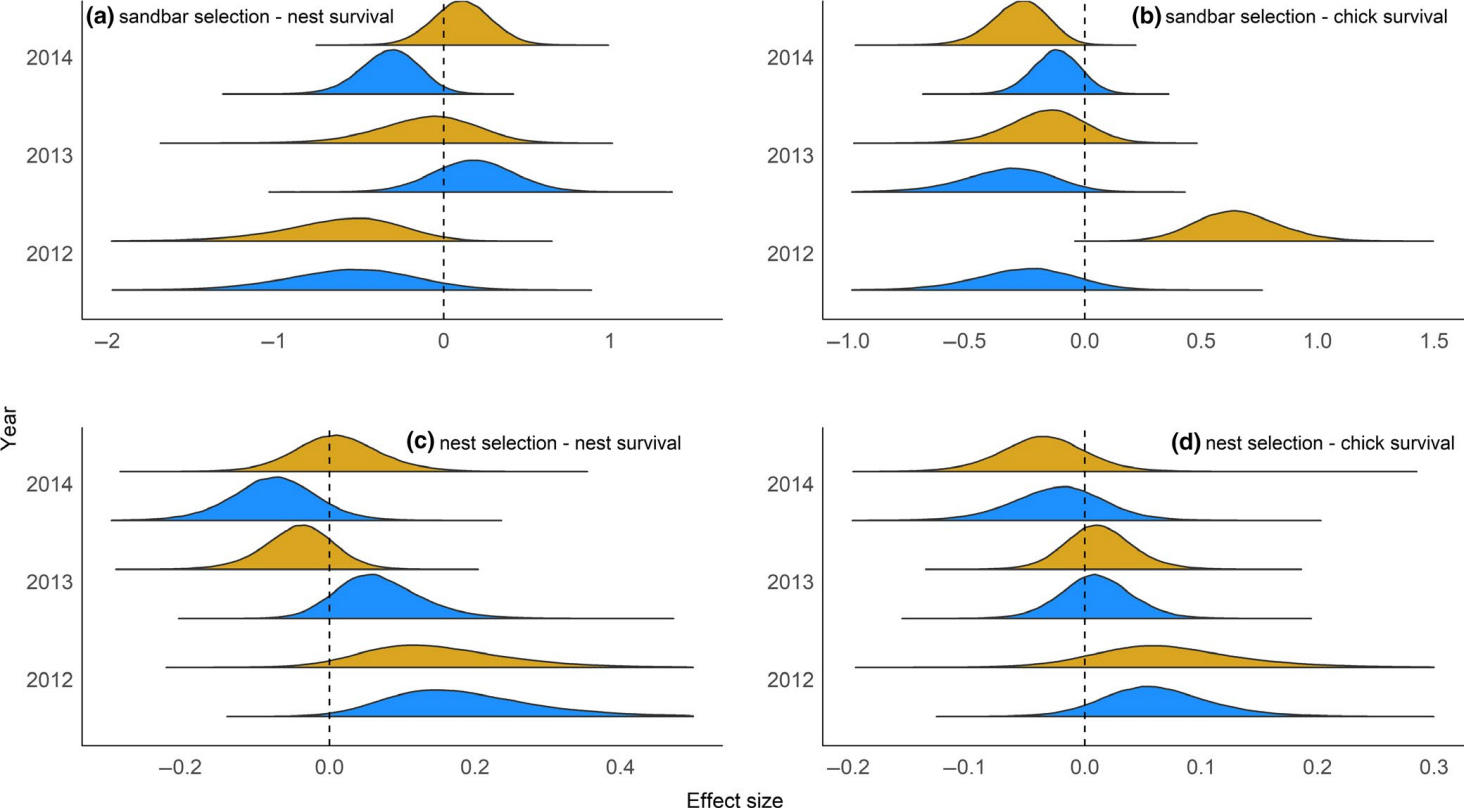
Consequences of sandbar scale selection (a, b) and nest‐site scale selection (c, d) on daily nest survival (a, c) and daily chick survival (b, d) of least terns (*Sternula antillarum*; goldenrod) and piping plovers (*Charadrius melodus*; blue) on the Missouri River (2012–2014). The estimates were derived from an integrated habitat selection and nest and chick survival model. Note that the scale of the x‐axes vary among plots

**Figure 6:**
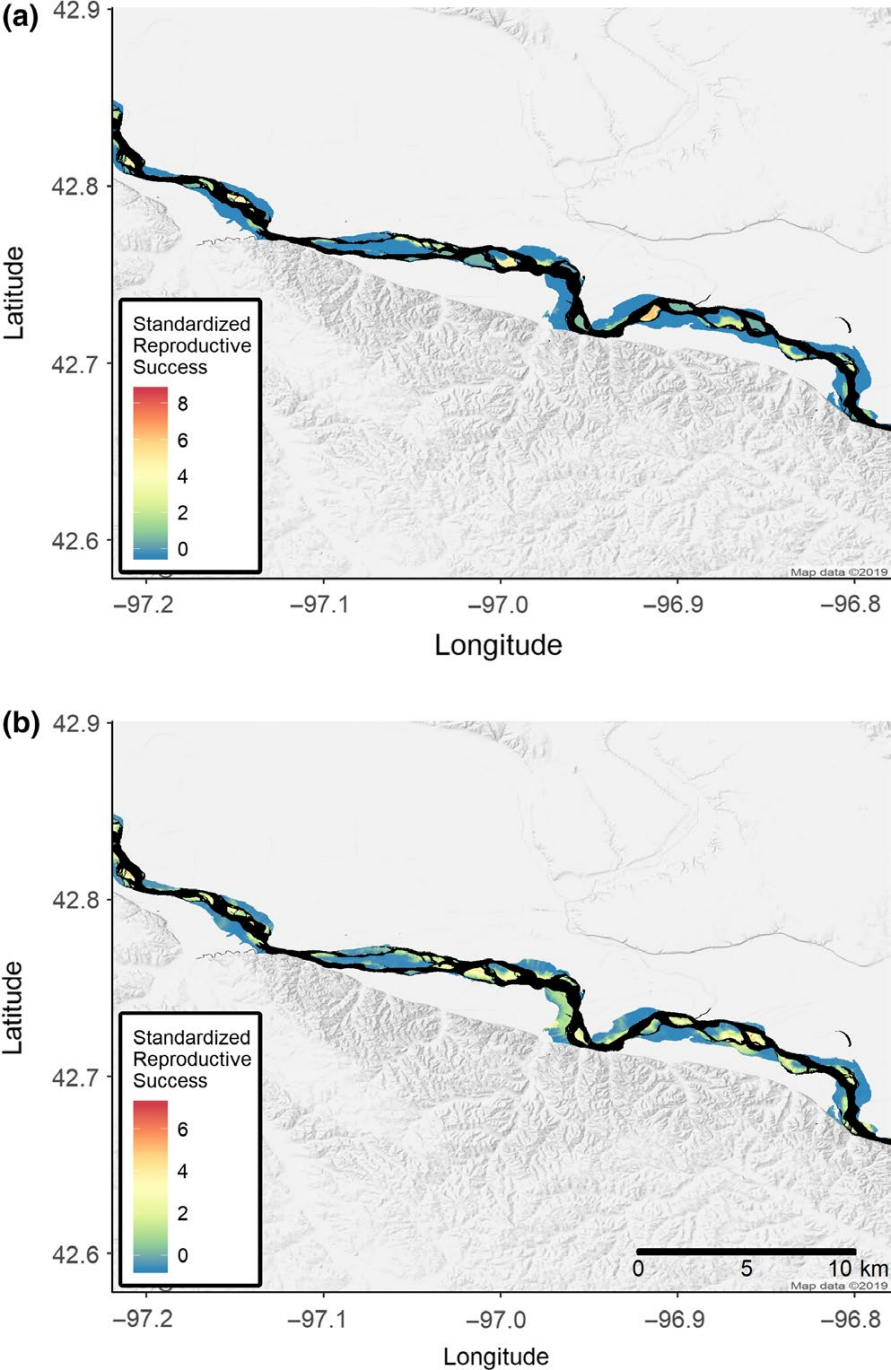
Standardized predicted reproductive success for least terns (*Sternula antillarum*; a) and piping plovers (*Charadrius melodus*; b) in 2012 from an integrated habitat selection model that assessed the effect of sandbar and nest‐site selection on both nest success and chick survival. Standardized reproductive success was the product of the probability of a location being occupied, the predicted nest success, and predicted chick survival, which was standardized for comparison. Blue hues indicate lower reproductive success, while red hues indicate higher reproductive success. Black indicates water

Sandbar selection had either a negative effect or no appreciable effect on daily nest survival for both species in all years. In particular, plover sandbar selection appeared to have a negative impact on nest survival in 2012 (−0.57 ± 0.39, *f* = 0.94) and 2014 (−0.33 ± 0.18, *f* = 0.97), whereas it only was negative in 2012 for terns (−0.63 ± 0.36, *f* = 0.98; Figure 5). Sandbar selection only had a positive effect on tern chick survival in 2012 (0.66 ± 0.18, *f* = 1.00), decreasing through the study (2013: −0.16 ± 0.17, *f* = 0.84 and 2014: −0.28 ± 0.12, *f* = 0.99), while sandbar selection had a consistent negative effect on plover chick survival (2012: −0.26 ± 0.22, *f* = 0.89, 2013: −33 ± 0.20, *f* = 0.97, and 2014: −0.12 ± 0.10, *f* = 0.88; Figure 5). The predicted effects of nest‐site selection on both nest success and chick survival were lower magnitude than those of sandbar selection, but they appeared consistent across species and years, such that nest‐site selection in 2012 had a positive impact on nest survival and chick survival for both terns (nest success: 0.15 ± 0.10, *f* = 0.95 and chick survival: 0.07 ± 0.07, *f* = 0.89) and plovers (nest success: 0.18 ± 0.10, *f* = 0.99 and chick survival: 0.06 ± 0.05, *f* = 0.93), and this trended toward a negative or no effect by 2014 (all *f* < 0.91, Figure 5). The differences between the species in habitat selection, nest success, and chick survival are evident when predicted; standardized reproductive success is projected onto the habitat (Figure 6).

## 4 DISCUSSION

Both least terns and piping plovers selected sandbars and nest sites within those sandbars that could reduce the chances of predation and flooding, and nest sites that would improve foraging opportunities for plover chicks. For chick survival, these decisions led to improved outcomes for both terns and plovers early in the study, but that diminished through time. The pattern for nest survival was less clear, suggesting that tern selection of sandbar and nest sites had less of a positive effect on their reproductive output than plovers.

### 4.1 Congruence between selection and success

The fact that selection was only partially predictive of success was perhaps not surprising. Both plovers and terns experienced exceptionally high nest and chick survival in the years following the floods (Hunt et al., 2018; Nefas et al., 2018), suggesting that predation and flooding were of little concern or that the densities were low enough that all birds were able to select relatively good habitat in this environment. It is unlikely that habitat was limiting the population immediately after the flooding (Robinson et al., 2019), and thus, it is possible that there were few if any birds that had to select marginal habitat. Similarly, cavity‐nesting species, which tend to have higher nesting success overall (Martin & Li, 1992), generally had the least congruence between selection and success among groups of birds, presumably because the pressure to select better habitat was less at such high success rates.

In their review of congruence in habitat selection studies and success, Chalfoun and Schmidt (2012) found that only 6.4% of studies showed complete congruence, 37.2% showed partial congruence, and 56.4% of studies showed no congruence at all, citing a series of anthropogenic, methodological, and ecological hypotheses for this pattern. Some of these mismatches were hypothesized to be related to anthropogenic changes in habitat that alter the relationship between habitat cues and the resulting fitness. For example, piping plovers nesting on Lake Sakakawea, a managed reservoir on the upper stretches of the Missouri River, chose nesting locations that led to higher than expected flooding of nests (Anteau et al., 2012), presumably because water fluctuations in the reservoir no longer resemble historical and ecological flows or selection for anti‐predator behavior is stronger than that against flooding, which may only exert selective pressure occasionally. Nest flooding was rare in the post‐flood population (Hunt et al., 2018), but it can severely affect certain populations in some years (Catlin et al., 2015). Sandbar height is positively correlated with the height of the water during a flood (Catlin et al., 2010), and the 2011 flood was the highest recorded since the closing of the dams (Hunt et al., 2018). Thus, it is possible that much of the available nesting habitat in this study was high enough that flooding was not an issue in these years, though plovers and terns would still select habitat to lessen the chances of flooding.

Both plovers and terns are relatively long‐lived species where chick survival tends to contribute more to population growth than nest survival (Catlin et al., 2016; Kirsch, 1996). Thus, we might expect to see more concordance between selection and chick survival since it is the more important value for fitness. Indeed, our results did show congruence between selection and chick survival in year 1 of our study, though it tended to decrease through time (Figure 6). Piping plovers have remarkably high site fidelity from 1 year to the next (Catlin et al., 2015; Friedrich, Hunt, Catlin, & Fraser, 2015), dispersing <200 m between years on average, such that early selection decisions can have lifelong consequences. However, as piping plover productivity generally declines through time because of vegetation growth or erosion of sandbars without some intervention (Hunt et al., 2018), we speculate that an increase in discordance between habitat selection and chick survival may be a natural process. Least terns also show moderate to high site fidelity, though less than plovers (Atwood & Massey, 1988; Renken & Smith, 1995), and colony locations were regular but variable sizes during our study (D. Catlin, personal observation). If flooding reduced the number of and cover for mammalian and avian predators on sandbars, but they continued to colonize through time, selection of a presumably good site early with high site fidelity could alter the effect on success.

The colonial nesting strategy exhibited by least terns may also have influenced the strength of a relationship between habitat selection and fitness. First, the selection of a nest site would most likely be an outcome of decision‐making process, where habitat as well as conspecific abundance or dominance structure would be considered. If the quality of the conspecific neighborhood influences fitness tangentially to the habitat characteristics associated with a particular area, observed patterns in habitat use may not fully describe selection. Given that the vast majority of variance in what was deemed suitable habitat for terns occurred at the sandbar scale, it is likely that fine‐scale nesting decisions within a sandbar were influenced by other factors in addition to local habitat characteristics. Second, given that geographically proximate areas are generally more similar to each other in habitat characteristics than are more distant areas, the habitat characteristics of individuals of a colonial nesting species may be more homogenous than in species that exhibit stronger territorial behavior. Increasingly, homogenous conditions would innately reduce the power to observe a relationship between habitat and fitness.

Nest and chick survival only are part of an individual's fitness, and other factors such as survival of the adults likely also contribute to a bird's selection of nesting habitat, further complicating the detection of fitness benefits (Chalfoun & Schmidt, 2012). Although we did not explicitly look at adult survival relative to habitat selection, avoidance of vegetative cover (Fraser & Catlin, 2019) and perch trees could contribute to adult survival, particularly raptor perch trees as avian predation appeared to be the greatest observed cause of adult mortality at our site (Catlin, Felio, et al., 2011; Catlin et al., 2015). For piping plovers, adult survival has the largest effect among the population parameters on population growth (Larson, Ryan, & Murphy, 2002; McGowan, 2005; Plissner & Haig, 2000), which likely is similar for interior least terns given their long life span and limited annual fecundity. Thus, we may expect the congruence between reproductive output and selection to be clouded somewhat by decisions that will optimize adult survival at the cost of lost reproductive output. We know from other studies that plovers will forego breeding in some years when the risk to their own survival may be high, but there is a complex interplay between breeding and survival (Weithman et al., 2017). Failure to detect a relationship between success and selection, therefore, could be a result of unmeasured fitness correlates that obscure relationships in some years.

### 4.2 Differences between the species

Although there are key differences in their life‐history characteristics (e.g., precocial vs. altricial, insectivorous vs. piscivorous), the two species experience similar pressures from predators and flooding. Thus, it would stand to reason that their selection would be similar with perhaps the exception of proximity of foraging (i.e., wet sand), but even that has some similarities since least tern adults tended to return to the wetted, moist sand with fish for young (D. Catlin, personal observation). Although there have been relatively few studies of nest selection for least terns, they tend to select open, sandy areas, often near inlets on the coast or on mid‐channel sandbars on prairie rivers (Burger & Gochfeld, 1990; Kirsch, 1996), which aligns with the more extensive work done to assess habitat suitability for piping plovers (Catlin, Fraser, et al., 2011; Cohen et al., 2009; Prindiville‐Gaines & Ryan, 1988). Least terns are colonial (Thompson et al., 1997), although less so in the Great Plains than on the Atlantic Coast (D. Catlin, personal observation), which could have contributed to the relatively stronger signal for selection in terns than plovers (i.e., larger selection coefficients, Table 2).

At the sandbar scale, plover sandbars were farther from the bank, in narrower stretches, and closer to cover than would be expected randomly, and tern sandbars were closer to the bank, in wider sections, but farther from cover and tended to be islands rather than bank‐connected bars (Figure 3). Piping plover adults, chicks, and fledglings preferentially forage in backwater channels (Le Fer, Fraser, & Kruse, 2008) similar to the low wave energy bayside mudflats that they prefer on the Atlantic Coast (Cohen et al., 2009), but mid‐channel island sandbars far from the bank often have deep channels on either side of them with little or no wetted sand on their peripheries. This need for wetted habitat could explain the differences we saw relative to the river width and island versus point bar parameters, as bank‐connected sandbars would likely have more wet sand than mid‐channel islands. For terns, which do not forage on moist sand, avoidance of cover and potential predators likely had a greater effect on their selection than plovers.

At the nest scale, with the exception of magnitude, plovers and terns used similar cues to select habitat (Figure 4), suggesting that they experience similar predation and flooding pressures at that scale. From our hypotheses, it is unclear why least terns nested nearer to wet sand than random and that the effect size was larger than for plovers that regularly display this behavior, presumably to optimize chick growth and survival (Cohen et al., 2009; Walker et al., 2019). It is possible that these locations facilitate feeding of chicks as mentioned above, but further research into these unexpected findings will be needed to move beyond speculation.

## 5 CONCLUSIONS

In general, terns and plovers made similar decisions when selecting sandbars and nest sites, decisions which should contribute to less predation and flooding, and to a degree, better foraging opportunities for plover chicks. Failure to detect congruence between these selective factors and reproductive output is common and found in over half of the studies that attempt to make those links (Chalfoun & Schmidt, 2012). Despite the difficulties in identifying the fitness benefits to selection, continued study of the effect of habitat selection on fitness is needed to understand the complex relationships and improve selection theory.

In this study, we present an integrated habitat selection and fitness model that can be modified further to accommodate a range of species and fitness correlates (e.g., adult survival). Continued application of similar models will help advance our knowledge of selection and its evolutionary underpinning. Ultimately how and why an animal selects its breeding habitat is of paramount importance in ecology and conservation. Thus, enhanced understanding of these factors will contribute to the conservation of imperiled species.

## CONFLICT OF INTEREST

None declared.

## AUTHOR CONTRIBUTIONS

DC, JDF, and SMK designed and administered all activities under this project. DC, KLH, and MJF collected the data associated with this manuscript. DG, DC, and MJF designed the analysis and developed the theoretical underpinnings of this study. DG and DC analyzed the data. All authors helped draft and edit the manuscript, and all approved of the final draft.

## Data Availability

All data can be found on figshare, https://doi.org/10.6084/m9.figshare.9992333. Model code is in Appendix S1.

## References

[ece35834-bib-0001] Amat, J. A. , & Masero, J. A. (2004). How Kentish plovers, *Charadrius* *alexandrinus*, cope with heat stress during incubation. Behavioral Ecology and Sociobiology, 56, 26–33. 10.1007/s00265-004-0758-9

[ece35834-bib-0002] Anteau, M. , Shaffer, T. , Sherfy, M. , Sovada, M. , Stucker, J. , & Wiltermuth, M. (2012). Nest survival of piping plovers at a dynamic reservoir indicates an ecological trap for a threatened population. Oecologia, 170, 1167–1179. 10.1007/s00442-012-2384-y 22700065

[ece35834-bib-0003] Atwood, J. L. , & Massey, B. W. (1988). Site fidelity of least terns in California. Condor, 90, 389–394. 10.2307/1368567

[ece35834-bib-0004] Boyce, M. S. , & McDonald, L. L. (1999). Relating populations to habitats using resource selection functions. Trends in Ecology & Evolution, 14, 268–272. 10.1016/S0169-5347(99)01593-1 10370262

[ece35834-bib-0005] Braden, G. T. , McKernan, R. L. , & Powell, S. M. (1997). Association of within‐territory vegetation characteristics and fitness components of California Gnatcatchers. The Auk, 114, 601–609. 10.2307/4089279

[ece35834-bib-0006] Brooks, S. P. , & Gelman, A. (1998). General methods for monitoring convergence of iterative simulations. Journal of Computational and Graphical Statistics, 7, 434–455.

[ece35834-bib-0007] Burger, J. (1987). Physical and social determinants of nest‐site selection in piping plover in New‐Jersey. Condor, 89, 811–818. 10.2307/1368529

[ece35834-bib-0008] Burger, J. , & Gochfeld, M. (1990). Nest site selection in least terns (*Sterna * *antillarum*) in New‐Jersey and New‐York. Colonial Waterbirds, 13, 31–40. 10.2307/1521418

[ece35834-bib-0009] Catlin, D. H. , Felio, J. H. , & Fraser, J. D. (2011). Effect of great horned owl trapping on chick survival in piping plovers. Journal of Wildlife Management, 75, 458–462. 10.1002/jwmg.56

[ece35834-bib-0010] Catlin, D. H. , Felio, J. H. , & Fraser, J. D. (2013). Effects of water discharge on fledging time, growth, and survival of piping plovers on the Missouri River. Journal of Wildlife Management, 77, 525–533. 10.1002/jwmg.494

[ece35834-bib-0011] Catlin, D. H. , Fraser, J. D. , & Felio, J. H. (2015). Demographic responses of piping plovers to habitat creation on the Missouri River. Wildlife Monographs, 192, 1–42. 10.1002/wmon.1016

[ece35834-bib-0012] Catlin, D. H. , Fraser, J. D. , Felio, J. H. , & Cohen, J. B. (2011). Piping plover habitat selection and nest success on natural, managed, and engineered sandbars. Journal of Wildlife Management, 75, 305–310.

[ece35834-bib-0013] Catlin, D. H. , Jacobson, R. B. , Sherfy, M. H. , Anteau, M. J. , Felio, J. H. , Fraser, J. D. , … Stucker, J. H. (2010). Discussion of "Natural hydrograph of the Missouri River near Sioux City and the least tern and piping plover" by Donald Jorgensen. Journal of Hydrological Engineering, 15, 1076–1078.

[ece35834-bib-0014] Catlin, D. H. , Zeigler, S. L. , Brown, M. B. , Dinan, L. R. , Fraser, J. D. , Hunt, K. L. , & Jorgensen, J. G. (2016). Metapopulation viability of an endangered shorebird depends on dispersal and human‐created habitats: Piping plovers (*Charadrius* *melodus*) and prairie rivers. Movement Ecology, 4, 6 10.1186/s40462-016-0072-y 26981249 PMC4791857

[ece35834-bib-0015] Chalfoun, A. D. , & Schmidt, K. A. (2012). Adaptive breeding‐habitat selection: Is it for the birds? The Auk, 129, 589–599. 10.1525/auk.2012.129.4.589

[ece35834-bib-0016] Clark, R. G. , & Shutler, D. (1999). Avian habitat selection: Pattern from process in nest‐site use by ducks? Ecology, 80, 272–287. 10.1890/0012-9658(1999)080[0272:AHSPFP]2.0.CO;2

[ece35834-bib-0017] Cohen, J. , Houghton, L. , & Fraser, J. (2009). Nesting density and reproductive success of piping plovers in response to storm‐ and human‐created habitat changes. Wildlife Monographs, 173, 1–24. 10.2193/2007-553

[ece35834-bib-0018] Elith, J. , & Leathwick, J. R. (2009). Species distribution models: Ecological explanation and prediction across space and time. Annual Review of Ecology Evolution and Systematics, 40, 677–697. 10.1146/annurev.ecolsys.110308.120159

[ece35834-bib-0019] Elliott‐Smith, E. , & Haig, S. M. (2004). Piping plover (*Charadrius**melodus*) In Poole A. (Ed.), The birds of North America online. Ithaca, NY: Cornell Lab of Ornithology 10.2173/bna.2

[ece35834-bib-0020] Espie, R. H. M. , Brigham, R. M. , & James, P. C. (1996). Habitat selection and clutch fate of Piping Plovers (*Charadrius* *melodus*) breeding at Lake Diefenbaker, Saskatchewan. Canadian Journal of Zoology, 74, 1069–1075.

[ece35834-bib-0021] Fletcher, K. , Aebischer, N. J. , Baines, D. , Foster, R. , & Hoodless, A. N. (2010). Changes in breeding success and abundance of ground‐nesting moorland birds in relation to the experimental deployment of legal predator control. Journal of Applied Ecology, 47, 263–272. 10.1111/j.1365-2664.2010.01793.x

[ece35834-bib-0022] Fraser, J. D. , & Catlin, D. H. (2019). Habitat ecology and conservation of Charadrius plovers In Colwell M. A., & Haig S. M. (Eds.), The population ecology and conservation of Charadrius plovers (pp. 217–243). Boca Raton, FL: CRC Press.

[ece35834-bib-0023] Friedrich, M. J. , Hunt, K. L. , Catlin, D. H. , & Fraser, J. D. (2015). The importance of site to mate choice: Mate and site fidelity in Piping Plovers. The Auk, 132, 265–276. 10.1642/AUK-14-100.1

[ece35834-bib-0024] Gaines, E. P. , & Ryan, M. R. (1988). Piping plover habitat use and reproductive success in North‐Dakota. Journal of Wildlife Management, 52, 266–273. 10.2307/3801233

[ece35834-bib-0025] Gibson, D. , Blomberg, E. J. , Atamian, M. T. , & Sedinger, J. S. (2017). Weather, habitat composition, and female behavior interact to modify offspring survival in Greater Sage‐Grouse. Ecological Applications, 27, 168–181. 10.1002/eap.1427 28052504

[ece35834-bib-0026] Gomez‐Serrano, M. A. , & Lopez‐Lopez, P. (2014). Nest site selection by Kentish plover suggests a trade‐off between nest‐crypsis and predator detection strategies. PLoS ONE, 9, e107121 10.1371/journal.pone.0107121 25208045 PMC4160202

[ece35834-bib-0027] Greenberg, R. , Elphick, C. S. , Nordby, J. C. , Gjerdrum, C. , Spautz, H. , Shriver, G. , … Winter, M. (2006). Flooding and predation: Trade‐offs in the nesting ecology of tidal‐marsh sparrows. Studies in Avian Biology, 32, 96–109.

[ece35834-bib-0028] Guilherme, J. L. , Burnside, R. J. , Collar, N. J. , & Dolman, P. M. (2018). Consistent nest‐site selection across habitats increases fitness in Asian Houbara. The Auk, 135, 192–205. 10.1642/AUK-17-156.1

[ece35834-bib-0029] Hanski, I. (2011). Habitat loss, the dynamics of biodiversity, and a perspective on conservation. Ambio, 40, 248–255. 10.1007/s13280-011-0147-3 21644453 PMC3357798

[ece35834-bib-0030] Hunt, K. L. , Fraser, J. D. , Friedrich, M. J. , Karpanty, S. M. , & Catlin, D. H. (2018). Demographic response of an imperiled shorebird suggests that engineered habitat restoration is no match for natural riverine processes. Condor, 120, 149–165.

[ece35834-bib-0031] Johnson, M. , & Oring, L. W. (2002). Are nest exclosures an effective tool in plover conservation? Waterbirds, 25, 184–190. 10.1675/1524-4695(2002)025[0184:ANEAET]2.0.CO;2

[ece35834-bib-0032] Jones, J. (2001). Habitat selection studies in avian ecology: A critical review. The Auk, 118, 557–562. 10.1093/auk/118.2.557

[ece35834-bib-0033] Kéry, M. , & Schaub, M. (2012). Bayesian population analysis using WinBUGS: A hierarchical perspective. Waltham, Massachusetts, USA: Academic Press.

[ece35834-bib-0034] Kirsch, E. M. (1996). Habitat selection and productivity of least terns on the lower Platte River, Nebraska. Wildlife Monographs, 132, 5–48.

[ece35834-bib-0035] Kotliar, N. B. , & Burger, J. (1986). Colony site selection and abandonment by least terns *Sterna * *antillarum* in New‐Jersey, USA. Biological Conservation, 37, 1–21. 10.1016/0006-3207(86)90031-5

[ece35834-bib-0036] Kruse, C. D. , Higgins, K. F. , & Lee, B. A. V. (2001). Influence of predation on piping plover, *Charadrius* *melodus*, and least tern, *Sterna * *antillarum*, productivity along the Missouri River in South Dakota. Canadian Field‐Naturalist, 115, 480–486.

[ece35834-bib-0037] Larson, M. A. , Ryan, M. R. , & Murphy, R. K. (2002). Population viability of piping plovers: Effects of predator exclusion. Journal of Wildlife Management, 66, 361–371. 10.2307/3803169

[ece35834-bib-0038] Le Fer, D. , Fraser, J. D. , & Kruse, C. D. (2008). Piping plover foraging‐site selection on the Missouri River. Waterbirds, 31, 587–592.

[ece35834-bib-0039] Loegering, J. P. , & Fraser, J. D. (1995). Factors affecting piping plover chick survival in different brood‐rearing habitats. Journal of Wildlife Management, 59, 646–655. 10.2307/3801940

[ece35834-bib-0040] Martin, T. E. (1988). Processes organizing open‐nesting bird assemblages: Competition or nest predation? Evolutionary Ecology, 2, 37–50. 10.1007/BF02071587

[ece35834-bib-0041] Martin, T. E. , & Li, P. J. (1992). Life‐history traits of open‐nesting vs cavity‐nesting birds. Ecology, 73, 579–592.

[ece35834-bib-0042] McGowan, C. (2005). Managing the effect of incidental take on the population viability of endangered species. Dissertation. University of Missouri, Columbia.

[ece35834-bib-0043] Melvin, S. M. , Macivor, L. H. , & Griffin, C. R. (1992). Predator exclosures ‐ A technique to reduce predation at piping plover nests. Wildlife Society Bulletin, 20, 143–148.

[ece35834-bib-0044] Miller, D. A. , Grand, J. B. , Fondell, T. F. , & Anthony, R. M. (2007). Optimizing nest survival and female survival: Consequences of nest site selection for Canada Geese. Condor, 109, 769–780. 10.1093/condor/109.4.769

[ece35834-bib-0045] Murray, L. D. , & Best, L. B. (2014). Nest‐site selection and reproductive success of Common Yellowthroats in managed Iowa grasslands. Condor, 116, 74–83. 10.1650/CONDOR-13-047-R1.1

[ece35834-bib-0046] Nefas, S. M. , Hunt, K. L. , Fraser, J. D. , Karpanty, S. M. , & Catlin, D. H. (2018). Least Tern (*Sternula* *antillarum*) nest success and chick survival on the Missouri River following historic flooding. Wilson Journal of Ornithology, 130, 371–376.

[ece35834-bib-0047] Orians, G. H. , & Wittenberger, J. F. (1991). Spatial and temporal scales in habitat selection. American Naturalist, 137, S29–S49. 10.1086/285138

[ece35834-bib-0048] Plissner, J. , & Haig, S. (2000). Viability of piping plover *Charadrius* *melodus* metapopulations. Biological Conservation, 92, 163–173. 10.1016/S0006-3207(99)00050-6

[ece35834-bib-0049] Plummer, M. (2003). A program for analysis of Bayesian graphical models using Gibbs sampling In 3rd International Workshop on Distributed Statistical Computing (DCS2003), Vienna, Austria.

[ece35834-bib-0050] Prindiville‐Gaines, E. P. , & Ryan, M. R. (1988). Piping plover habitat use and reproductive success in North Dakota. Journal of Wildlife Management, 52, 266–273. 10.2307/3801233

[ece35834-bib-0051] R Core Team (2012). R: A language and environment for statistical computing. Vienna, Austria: R Foundation for Statistical Computing Retrieved from http://www.R-project.org/

[ece35834-bib-0052] Renken, R. B. , & Smith, J. W. (1995). Interior least tern site fidelity and dispersal. Colonial Waterbirds, 18, 193–198. 10.2307/1521480

[ece35834-bib-0053] Robinson, S. G. , Fraser, J. D. , Catlin, D. H. , Karpanty, S. M. , Altman, J. M. , Boettcher, R. , … Wilke, A. (2019). Irruptions: Evidence for breeding season habitat limitation in Piping Plovers (*Charadrius* *melodus*). Avian Conservation and Ecology, 14, 19.

[ece35834-bib-0054] Rotella, J. J. , Taper, M. L. , & Hansen, A. J. (2000). Correcting nesting‐success estimates for observer effects: Maximum‐likelihood estimates of daily survival rates with reduced bias. The Auk, 117, 92–109. 10.1093/auk/117.1.92

[ece35834-bib-0055] Shaffer, T. L. (2004). A unified approach to analyzing nest success. The Auk, 121, 526–540. 10.1642/0004-8038(2004)121[0526:AUATAN]2.0.CO;2

[ece35834-bib-0056] Sherfy, M. H. , Stucker, J. H. , & Buhl, D. A. (2012). Selection of nest‐site habitat by interior least terns in relation to sandbar construction. Journal of Wildlife Management, 76, 363–371. 10.1002/jwmg.301

[ece35834-bib-0057] Sidle, J. G. , Carlson, D. E. , Kirsch, E. M. , & Dinan, J. J. (1992). Flooding: Mortality and habitat renewal for Least Terns and Piping Plovers. Colonial Waterbirds, 15, 132–136. 10.2307/1521363

[ece35834-bib-0058] Smith, P. A. , Gilchrist, H. G. , & Smith, J. N. M. (2007). Effects of nest habitat, food, and parental behavior on shorebird nest success. Condor, 109, 15–31. 10.1093/condor/109.1.15

[ece35834-bib-0059] Southwood, T. R. E. (1977). Habitat, templet for ecological strategies ‐ Presidential‐address to British‐Ecological‐Society, 5 January 1977. Journal of Animal Ecology, 46, 337–365.

[ece35834-bib-0060] Stokes, D. L. , & Boersma, P. D. (1998). Nest‐site characteristics and reproductive success in magellanic penguins (*Spheniscus* * magellanicus*). The Auk, 115, 34–49. 10.2307/4089109

[ece35834-bib-0061] Storey, A. E. , Montevecchi, W. A. , Andrews, H. F. , & Sims, N. (1988). Constraints on nest site selection ‐ A comparison of predator and flood avoidance in 4 species of marsh‐nesting birds (Genera, Catoptrophorus, Larus, Rallus, and Sterna). Journal of Comparative Psychology, 102, 14–20. 3365941 10.1037/0735-7036.102.1.14

[ece35834-bib-0062] Swaisgood, R. R. , Nordstrom, L. A. , Schuetz, J. G. , Boylan, J. T. , Fournier, J. J. , & Shemai, B. (2018). A management experiment evaluating nest‐site selection by beach‐nesting birds. Journal of Wildlife Management, 82, 192–201. 10.1002/jwmg.21342

[ece35834-bib-0063] Tan, L. X. L. , Buchanan, K. L. , Maguire, G. S. , & Weston, M. A. (2015). Cover, not caging, influences chronic physiological stress in a ground‐nesting bird. Journal of Avian Biology, 46, 482–488. 10.1111/jav.00625

[ece35834-bib-0064] Thompson, B. C. , Jackson, J. A. , Burger, J. , Hill, L. A. , Kirsch, E. M. , & Atwood, J. L. (1997). Least tern (*Sterna **antillarum*), version 2.0 In Poole A. F. & Gill F. B. (Eds.), Birds of North America. Ithaca, NY, USA: Cornell Lab of Ornithology 10.2173/bna.290

[ece35834-bib-0065] Walker, K. M. , Fraser, J. D. , Catlin, D. H. , Ritter, S. J. , Robinson, S. G. , Bellman, H. A. , … Papa, S. T. (2019). Hurricane Sandy and engineered response created habitat for a threatened shorebird. Ecosphere, 10, e02771 10.1002/ecs2.2771

[ece35834-bib-0066] Weithman, C. E. , Gibson, D. , Hunt, K. L. , Friedrich, M. J. , Fraser, D. F. , Karpanty, S. M. , & Catlin, D. H. (2017). Senescence and carryover effects of reproductive performance influence migration, condition, and breeding propensity in a small shorebird. Ecology and Evolution, 7, 11044–11056. 10.1002/ece3.3533 29299280 PMC5743479

[ece35834-bib-0067] Westerskov, K. (1950). Methods for determining the age of game bird eggs. Journal of Wildlife Management, 14, 56–67. 10.2307/3795978

