# Supplementary material for: Habitat selection and potential fitness consequences of two early‐successional species with differing life‐history strategies
Source: Ecol Evol. 2019 Nov 19;9(24):13966–78. doi: 10.1002/ece3.5834 (PMC6953654; doi:10.1002/ece3.5834)
Supplement: Supplementary file 1 [file ECE3-9-13966-s001.docx]

**Appendix 1:**

Habitat selection and potential fitness consequences of two early-successional species with differing life-history strategies

Daniel Catlin^1^, Daniel Gibson, Meryl J. Friedrich, Kelsi L. Hunt, Sarah M. Karpanty, and James D. Fraser

Department of Fish and Wildlife Conservation, Virginia Tech, Blacksburg, VA 24060

^1^ Corresponding author email: dcatlin@vt.edu

sink("global.jags")

cat(

"model {

######################################################

# Sandbar scale nest site selection model

#in this step, the outcomes are 1:plover only,2:plover and tern, 3:unused

#covariates: 1. dist to tree, 2. dist to cover, 3. dist to bank, 4. river width

# 5. mod canopy, 6. low canopy, 7. area, 8. dry sand, 9. wet sand

# 10. island v point

######################################################

# Priors and constraints

for (k in 1:n.year){

b0[1,k] ~ dlogis(0,1)

b0[2,k] ~ dlogis(0,1)

b0[3,k] <- 0

}

# Model likelihood

for( i in 1:n.sandbar) {

use[i] ~ dcat(p[i, 1:n.outcomes])

sandbar.use[i,1] <- p[i,2] #lete only (lete and pipl bars = 2)

sandbar.use[i,2] <- 1 - p[i,3] #lete and pipl (lete and pipl = 2, pipl only =1, so all pipl = 1+2)

for (j in 1:n.outcomes){

log(p.l[i,j]) <- b0[j, year_sand[i]] + inprod(covars[i,], b1[j,])

p[i,j] <- p.l[i,j]/sum(p.l[i,1:n.outcomes])

}

}

# Prior for betas

for (c in 1:n.covariates){

b1[3,c] <-0

b1[1,c] ~ dnorm(0,0.001)

b1[2,c] ~ dnorm(0,0.001)

}

# Derive sandbar-level RSF coefficient

for (k in 1:n.predictions){

sandbar_selection[k] <- b0[species[k], year_s[k]] +

b1[species[k],1] * pred.covs[k,1] +

b1[species[k],2] * pred.covs[k,2] +

b1[species[k],3] * pred.covs[k,3] +

b1[species[k],4] * pred.covs[k,4] +

b1[species[k],5] * pred.covs[k,5] +

b1[species[k],6] * pred.covs[k,6] +

b1[species[k],7] * pred.covs[k,7] +

b1[species[k],8] * pred.covs[k,8] +

b1[species[k],9] * pred.covs[k,9] +

b1[species[k],10] * pred.covs[k,10]

}#k

############################################################################################

# Nest-scale nest site selection model

# Species - 1: LETE, 2: PIPL, yes, it switches between steps

# covariates: 1. dist to wet sand, 2. dist to floodplain, 3. dist to tree, 4. dist to cover, 5. river width,

# 6. dist to waterline, 7.dry sand (y/n),

############################################################################################

# Priors and constraints

for (m in 1:n.cov){

b.i[1,m] ~ dnorm(0,0.001)

b.i[2,m] ~ dnorm(0,0.001)

}

# Model likelihood

for (i in 1:N) {

joint.p[i] <- p.i[i] * sandbar.use[N_sand[i], species.i[i]]

use.i[i] ~ dbern(joint.p[i])

logit(p.i[i]) <- inprod(covars_ns[i,], b.i[species.i[i],])

}

# Derive individual-level RSF coefficient

for (n in 1:n.nests){

nest_selection[n] <-

b.i[N_SPECIES[n], 1] * pred_covs_ns[n,1] +

b.i[N_SPECIES[n], 2] * pred_covs_ns[n,2] +

b.i[N_SPECIES[n], 3] * pred_covs_ns[n,3] +

b.i[N_SPECIES[n], 4] * pred_covs_ns[n,4] +

b.i[N_SPECIES[n], 5] * pred_covs_ns[n,5] +

b.i[N_SPECIES[n], 6] * pred_covs_ns[n,6] +

b.i[N_SPECIES[n], 7] * pred_covs_ns[n,7]

}

############################################################################################

# Nest Survival model

# Species - 1: LETE, 2: PIPL

############################################################################################

#Priors and constraints

for(m in 1:nspecies){

for(k in 1:nyear){

intercept[m,k] ~ dlogis(0,1)

sandbar.beta[m,k] ~ dnorm(0,0.001)

nestsite.beta[m,k] ~ dnorm(0,0.001)

}

}

# Model likelihood

for(i in 1:nvisit){

eta[i]<- intercept[species_ns[i], year_ns[i]]

+ sandbar.beta[species_ns[i],year_ns[i]] * sandbar_selection[sandbar[i]] #sandbar selection

+ nestsite.beta[species_ns[i],year_ns[i]] * nest_selection[nest.id[i]] # nest-site selection

logit(phi[i])<-eta[i] #anti-logit to determine the daily survival rate

mu[i]<-pow(phi[i],interval[i]) #period survival is DSR raised to the interval

succ[i]~dbern(mu[i]) #the data is distributed as bernoulli with period survival as the mean

}

############################################################################################

# Chick Survival model - uses psi and q for phi and p bc phi and p was used earlier

############################################################################################

for (i in 1:nind){

for (t in 1:(n.occasions[cs_species[i]]-1)){

logit(psi[cs_species[i],i,t])<-

lmean.psi[cs_species[i],cs_year[i]]

+ agePsi[cs_species[i]]*(t-1)

+ beta_cs.sandbar[cs_species[i],cs_year[i]] * sandbar_selection[cs_sandbar[i]] #sandbar selection

+ beta_cs.nest[cs_species[i],cs_year[i]] * nest_selection[cs_nest.id[i]] # nest-site selection

logit(q[cs_species[i],i,t])<-lmean.q[cs_species[i]] + ageq[cs_species[i]]*(t-1)

}#t

}#i

for (n in 1:nspecies){

lmean.q[n] ~ dlogis(0,1) #prior for mean q logit

for (k in 1:nyear){

beta_cs.sandbar[n,k] ~ dnorm(0,0.001)

beta_cs.nest[n,k] ~ dnorm(0,0.001)

}#k

}#n

agePsi[1]<-0

agePsi[2] ~ dnorm(0,0.001) #prior for age effect on Psi

ageq[1] <-0

ageq[2] ~ dnorm(0,0.001)

#likelihood

for (i in 1:nind){

#Define latent state at first capture

z[i,f[i]]<-1

for (t in (f[i]+1):n.occasions[cs_species[i]]){

#State process

z[i,t] ~ dbern(mu1[i,t])

mu1[i,t]<-psi[cs_species[i],i,t-1]*z[i,t-1]

#observation process

y[i,t]~dbern(mu2[i,t])

mu2[i,t]<-q[cs_species[i],i,t-1]*z[i,t]

}#t

}#i

}

",fill = TRUE)

sink()
